# Gut microbiota in Immunoglobulin A Nephropathy: a Malaysian Perspective

**DOI:** 10.1186/s12882-021-02315-z

**Published:** 2021-04-22

**Authors:** Agni Nhirmal Kumar Sugurmar, Rozita Mohd, Shamsul Azhar Shah, Hui-min Neoh, Rizna Abdul Cader

**Affiliations:** 1 Department of Medicine, Universiti Kebangsaan Malaysia Medical Centre, Jalan Yaacob Latif, Bandar Tun Razak, 56000 Kuala Lumpur, Malaysia; 2 Department of Community Health, Universiti Kebangsaan Malaysia Medical Centre, Kuala Lumpur, Malaysia; 3 UKM Molecular Biology Institute, Kuala Lumpur, Malaysia; 4 ParkCity Medical Centre, No.2, Jalan Intisari Perdana, Desa ParkCity, Kuala Lumpur, 52200 Malaysia

**Keywords:** Gut microbiota, IgA nephropathy, Alpha diversity, Chronic kidney disease, Microbiome, Dysbiosis, *Firmicutes/Bacteroidetes* ratio

## Abstract

**Introduction:**

The alteration of the gut microbiome in the gut-kidney axis has been associated with a pro-inflammatory state and chronic kidney disease (CKD). A small-scaled Italian study has shown an association between the gut microbiome and Immunoglobulin A Nephropathy (IgAN). However, there is no data on gut microbiota in IgAN in the Asian population. This study compares the gut microbial abundance and diversity between healthy volunteers and Malaysian IgAN cohort.

**Methods:**

A comparative cross-sectional study was conducted involving biopsy-proven IgAN patients in clinical remission with matched controls in a Malaysian tertiary centre. Demographic data, routine blood and urine results were recorded. Stool samples were collected and their DNA was extracted by 16S rRNA gene sequencing to profile their gut microbiota.

**Results:**

Thirty-six IgAN patients (13 male; 23 female) with the mean age of 45.5 ± 13.4 years and median estimated glomerular filtration rate (eGFR) of 79.0 (62.1–92.2) mls/min/1.73m^2^ with median remission of 7 years were analysed and compared with 12 healthy controls (4 male; 8 female) with the mean age of 46.5 ± 13.5 years and eGFR of 86.5 (74.2–93.7) mls/min/1.73m^2^. Other demographic and laboratory parameters such as gender, ethnicity, body mass index (BMI), haemoglobin, serum urea and serum albumin were comparable between the two groups. There were no significant differences seen in the Operational Taxonomic Unit (OTU) and alpha diversity (Shannon index) between IgAN and healthy controls. Alpha diversity increased with increasing CKD stage (*p* = 0.025). Firmicutes/Bacteroidetes (F/B) ratio was low in both IgAN and healthy cohort. *Fusobacteria* phylum was significantly increased (*p* = 0.005) whereas *Euryarchaoeota* phylum was reduced (*p* = 0.016) in the IgAN group as compared to the control cohort.

**Conclusion:**

Although we found no differences in OTU and alpha diversity between IgAN in remission and control cohort, there were some differences between the two groups at phylum level.

**Supplementary Information:**

The online version contains supplementary material available at 10.1186/s12882-021-02315-z.

## Introduction

Immunoglobulin A Nephropathy (IgAN) also known as synpharingitic nephritis is the most prevalent form of primary glomerulonephritis (GN) worldwide [1]. This autoimmune disease tends to affect the youth and accounts for 23% of GN in Malaysia [2]. In certain European countries, the reported prevalence was > 30% [1]. The depicted local prevalence is underestimated as a histopathological examination is not done comprehensively for all suspected cases and the existence of subclinical or indolent IgAN [3].

IgAN is a disease of abnormal Immunoglobulin A (IgA); it forms immune complexes that deposit into the mesangium and capillary walls causing glomerular injury and glomerulonephritis that manifest as nephritic syndrome. Abnormal IgA is due to defective glycosylation process caused by galactose deficiency [4]. Accumulation of abnormal IgA will bind to antiglycan Immunoglobulin G and form immune complexes. The exact pathogenesis is still ambiguous, but numerous studies postulate the multi-hit theory and the role of genes as possible causes [5, 6].

IgA is mainly found in the mucosal layers of the gut, genitourinary, respiratory tract, saliva, breast milk and tears [7]. Contemporary studies demonstrate that the gut is not only responsible for most of IgA production but also utilises IgA for maintaining gut mucosal colonisation [8]. There are more than a 1000 different species identified in the human gut and this community is termed as gut microbiome [9]. These vast arrays of microbiomes are in a perpetual symbiotic relationship between one another and the host, providing trophic and protective functions [10] including the development of the metabolic system, maturation of the intestinal immune system and the catering of essential nutrients [11, 12]. However, gut microbiota is heavily influenced by diet, environmental factors and socioeconomics, along with host genotype and genetic predisposition [13, 14].

Dysbiosis of the gut microbiome is increasingly recognised to be associated with various medical conditions including chronic kidney disease (CKD) [15–18]. Vaziri et al. demonstrate that CKD was associated with lower diversity and richness of gut microbiota and attributed these to the elevated urea in CKD [18].

*Firmicutes* and *Bacteroidetes* are the major phyla representing the gut microbiome [19, 20]. Novel researches have emphasized on the *Firmicutes* to *Bacteroidetes* ratio – F/B ratio, as a marker of gut dysbiosis. Data on F/B ratio in IgAN is scarce. Various studies of other autoimmune and metabolic diseases have shown inconclusive F/B ratio. Studies found reduced F/B ratio in type 1 diabetes mellitus and systemic lupus erythematous [21]. In contrast, the F/B ratio was increased in psoriasis, a different form of autoimmune disease [22]. Other studies involving obesity [23–25] and multiple sclerosis patients showed inconsistent F/B ratio [26, 27]. These calls for more research to delineate the F/B ratio in IgAN. Additionally, the role of treating dysbiosis is also taking a forefront. In a mouse model study, reduced *Lactobacillus* was found in mice with lupus nephritis, and increasing *Lactobacillus* with caecal microbiota transplantation was associated with an improvement in both renal and overall survival [28].

The first data on gut microbiota in IgA was published in 2014 as De Angelis et al. demonstrated a lower density of microbes especially the *Clostridium, Enterococcus, Lactobacillus, Bifidobacterium* genera [29]. The F/B ratio, as well as *Firmicutes* and *Proteobacteria* phylum, were increased in the IgAN group compared to healthy controls. Another study on Henoch Schönlein Purpura found a low diversity and richness in the disease group and serum IgA levels exhibited a significant negative correlation with the genus *Bifidobacterium* [30].

Thus, we aim to study the association of IgAN and gut microbiota in Asians, bearing in mind that the frequency and severity of IgAN in Asian population is different from that of Caucasians [31]. Furthermore, Asians are genetically different with notable variations in geographical regions, climate, dietary habits and lifestyle compared to Europeans. Our primary objective was to compare gut microbiome profile of IgAN patients in remission with control group. Understanding the gut microbiota of Asian IgAN may someday facilitate targeted correction of dysbiosis and possibly improve renal and overall survival.

## Materials and methods

### Study design and participants

This was a comparative cross-sectional study involving biopsy-proven IgA nephropathy patients attending the nephrology clinic follow up at Universiti Kebangsaan Malaysia Medical Centre (UKMMC) from August 2019 to January 2020. We included patients with biopsy proven IgAN aged more than 18 years and in disease remission. We excluded patients with diabetes (type 1 and 2), liver diseases, autoimmune diseases, malignancies, gastrointestinal diseases, ischaemic heart diseases, pregnancy and patients with serum urea more than 20 mmol/L or end-stage renal diseases. We also excluded patients who have travelled abroad or were receiving antibiotics, immunosuppressant or probiotics in the past 3 months prior to our study. The control cohort consists of mostly healthy individuals, patients with stable hypertension disease on single antihypertensive and corrected obstructive uropathy. They were recruited for comparison and matched by age, gender, ethnicity and Body Mass Index (BMI).

Before enrolment all subjects provided written informed consent. All subjects that fulfilled the study criteria and consented were asked to keep a dietary diary to ensure no marked change in their usual dietary practices. Biochemical and clinical tests including faecal sample collection were performed in accordance with relevant guidelines and regulations, including Good Clinical Practice guidelines. Demographic data and disease history were recorded. Routine blood tests including full blood count, renal profile and liver function test, urinalysis and urine protein-creatinine index (UPCI) were done. Estimated Glomerular Filtration Rate (eGFR) was calculated based on the CKD-EPI 2009 equation [32]. Subjects were briefed on effective stool collection with minimal contamination during a consultation and via a pamphlet.

The study was approved by UKMMC research and ethics committee (FF-2019-352) and registered with National Medical Research Registry (NMRR-19-3331-51,504). This study was jointly funded by a grant from the Malaysian National Kidney Foundation and Universiti Kebangsaan Malaysia.

### Stool sampling and DNA extraction

Stool samples were taken at home and brought to the hospital within 6 h in cold storage.

Samples were stored at -80 °C in the laboratory. DNA extraction was performed using GeneAll Exgene™ Stool DNA kit (Cambio Ltd., Cambridge, England) as per manufacturer protocol. Concentration and quality of DNA extracts were monitored using Nanodrop Spectrophotometer (Nanodrop Technologies, Wilmington, Delaware).

### Gene sequencing - 16S rRNA analysis

Following DNA extraction, 16S rRNA gene fragments were amplified from the extracted DNA. The gene-specific sequences used in the protocol targets the 16 s rDNA V3 and V4 region as published in the literature [33]. Illumina adapter overhang nucleotide sequences were added to the gene-specific sequences. The full-length primer sequences, using standard International Union of Pure and Applied Chemistry (IUPAC) nucleotide nomenclature, to follow the protocol targeting this region were:

16S Amplicon PCR Forward Primer = 5′

*TCGTCGGCAGCGTCAGATGTGTATAAGAGACAGCCTACGGGNGGCWGCAG*

16S Amplicon PCR Reverse Primer = 5′ *GTCTCGTGGGCTCGGAGATGTGTATAAGAGACAGGACTACHVGGGTATCTAATCC*

Forward overhang: 5′

*TCGTCGGCAGCGTCAGATGTGTATAAGAGACAG*- [locus-specific sequence]

Reverse overhang: 5′

*GTCTCGTGGGCTCGGAGATGTGTATAAGAGACAG*- [locus-specific sequence]

Reads from the sequencing were sorted by bcl2fastq2 software using unique barcodes. The barcode, linker, and primer sequences were then removed from the original sequencing reads. The merged reads containing two or more ambiguous nucleotides, those with a low-quality score (average score < 20), or reads shorter than 300 Base pair, were filtered out. Potential chimeric sequences were detected using the ChimeraSlayer r20110519.

The pre-processed reads from each sample were used to calculate the number of OTUs which was determined by clustering the sequences from each sample using a 97% sequence identity cut-off using Quantitative Insights Into Microbial Ecology (QIIME) software (v.1.8.0).

Taxonomic abundance was counted with RDP Classifier v2.11 using a confidence threshold of 0.8 derived from the pre-processed reads for each sample and NCBI Blast v2.2.28 following clustering by CD-HIT v4.6 using a 99% sequence identity with 80% read coverage cut-off [34, 35]. The microbial composition was normalized by dividing the value calculated from the taxonomy abundance count with the number of pre-processed reads for each sample. To measure the alpha diversity of each sample, the OTUs were analysed using the Shannon index, $H\hbox{'}=\hbox{-} {\sum}_{i=1}^S\left({p}_i\;\ln\;\left({p}_i\right)\right)$.

### Statistical analysis

All data presented in this study were analysed using SPSS software version 21 (IBM Inc., Chicago, IL, USA). The sample size was calculated based on the only available study describing gut microbiota and IgAN at the time, De Angelis et al. [29]. We required 8 experimental subjects and 8 control subjects to be able to reject the null hypothesis that the population means of the experimental and control groups are equal with probability (power) 0.8. The Type I error probability associated with this test of this null hypothesis is 0.05. We recruited more considering the possibility of dropouts and sampling error.

The rationale behind 3:1 ratio was due to a myriad of variables that may influence the gut microbiome. Statistical significance can be achieved by conventional 1:1 ratio but increasing the number participants in the disease group will reduce the weight of these unaccounted variables as well as reduce selection bias.

All data were tested for normality. Next, normally distributed data were analysed with mean and standard deviation while skewed distributed data were analysed with median and interquartile range (25–75%). Independent t-test and Mann Whitney test were used to compare the variables. In order to adjust for false discovery rate, we used the Bonferroni multiple comparison correction. Our adjusted *p* value / q value of 0.05 was considered statistically significant.

## Results

### Subject profiles and characteristics

We recruited a total of 36 IgAN patients with 12 controls. Both groups were comparable in their demographics parameters including age, gender, ethnic distribution, as well as BMI and eGFR. Biochemical parameters were also comparable except for UPCI that was higher in the IgAN group and summarised in Table 1. The IgAN subjects were in clinical remission for at least 1 year with a median duration of remission being 7 years (IQR 4–13). Majority of our patients had only 1 relapse and all treated as per KDIGO guideline with antiproteinuric agents, namely Angiotensin-Converting Enzyme inhibitor (ACE-i) or Angiotensin II Receptor Blocker (ARB) [36]. All 36 patients (100%) in our IgAN cohort were treated with either ACE-i or ARB and 23 patients (63.9%) were on fish oil therapy. 7 out of 12 (58%) control patients were on ACE-i/ARB for their hypertension. All these patients have good hypertension control on ACE-i/ARB.

**Table 1:** Demographic and laboratory data in IgAN patients and healthy controls

	IgAN cohort (***n*** = 36)	Healthy cohort (***n*** = 12)	***P*** value
	Mean **+** SD/ Median (IQR)	Mean **+** SD/ Median (IQR)	
**Age (years)**	45.5 + 13.4	46.5 + 13.5	0.814
**Gender [n, (%)]**			
Male	13 (36%)	4 (33%)	0.865
Female	23 (64%)	8 (67%)	
**Race [n, (%)]**			
Malay	17 (47.2%)	6 (50%)	0.871
Chinese	19 (52.8%)	6 (50%)	
**BMI (kg/m**^**2**^**)**	24.56 + 2.79	23.62 + 3.03	0.326
**Haemoglobin (g/dL)**	13.43 + 1.56	13.18 + 1.54	0.707
**Urea (mmol/L)**	4.7 (4.2–5.7)	4.0 (3.4–5.0)	0.072
**Creatinine (μmol/L)**	83.5 (62.3–116.5)	69.5 (59.3–84.3)	0.14
**eGFR (mls/min/1.73m**^**2**^**)**	79.0 (62.1–92.2)	86.5 (74.3–93.8)	0.248
**Albumin (g/L)**	41.39 + 3.36	42.33 + 1.97	0.245
**UPCI (g/mmol creatinine)**	0.050 (0.023–0.128)	0.01 (0.01–0.01)	< 0.001

*BMI* Body Mass Index, *eGFR* estimated Glomerular Filtration Rate based on CKD EPI 2009

*UPCI* Urine Protein-Creatinine Index

### Characterisation of intestinal microbiome

The number of reads in all 48 subjects by 16S rRNA V3 and V4 amplicon sequencing was 3,797,148. This final read number was obtained post trimming and quality control. The median for the number of reads for disease cohort was 87,125 (54272–99,417) as shown in Table 2.

**Table 2:** Gut microbiome analysis between the two groups

	IgAN cohort (***n*** = 36)	Control cohort (***n*** = 12)	***P***-value
	Mean **+** SD/ Median (IQR)	Mean **+** SD/ Median (IQR)	
**Number of reads**	87,125 (54273–99,417)	84,188 (74645–93,178)	0.849
**Number of OTUs**	10,080.64 + 3018.92	11,015.33 + 2163.45	0.328
**Shannon index**	4.986 + 0.508	5.046 + 0.595	0.739

There were no significant differences in OTUs or alpha diversity measured in the Shannon index between IgAN and controls. Further analysis indicated no relationship between the number of OTUs and Shannon index with ethnicity, gender or BMI. However, the IgAN cohort had significant association of alpha diversity with eGFR < 60 mls/min/1.73m^2^, *p* = 0.025. The alpha diversity increased with the reduction of eGFR. These findings were not found in the control cohort.

**Fig. 1:**
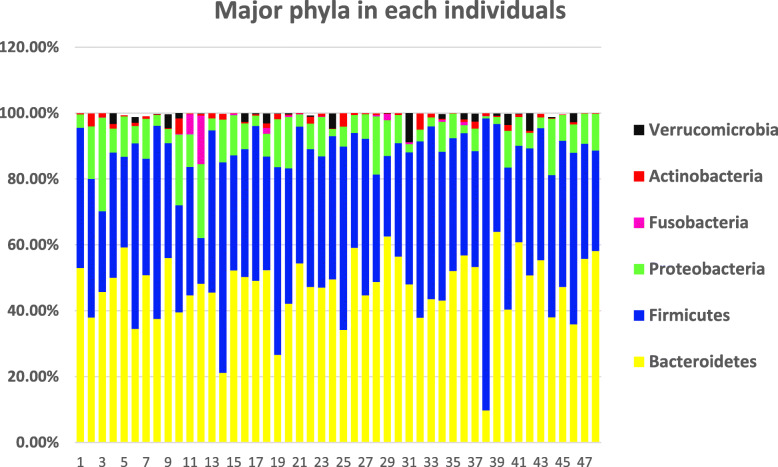
Bar plot of the composition of major phyla in each individual sample

**Fig. 2:**
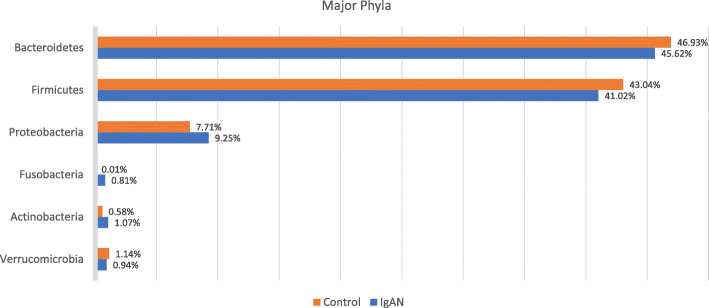
Phyla abundance between cohorts

A total of 24 phyla, 48 classes and 685 genera were identified. Six major phyla dominated the composition of the gut microbiota – *Bacteroidetes, Firmicutes, Proteobacteria* and to a lesser extent *Verrucomicrobia, Actinobacteria* and *Fusobacteria*. These phyla are shown in each individual subject in Fig. 1. Figure 2 demonstrates the composition of these phyla between the two cohorts.

IgAN cohort had an increased abundance in *Proteobacteria, Fusobacteria* and *Actinobacteria* but reduced *Firmicutes, Bacteriodetes* and *Verrucomicrobia* when compared with controls as shown in Fig. 2. The *Firmicutes / Bacteroidetes* ratio (F/B ratio) was low in both our cohorts (0.87 in IgAN and 0.89 in healthy).

**Fig. 3:**
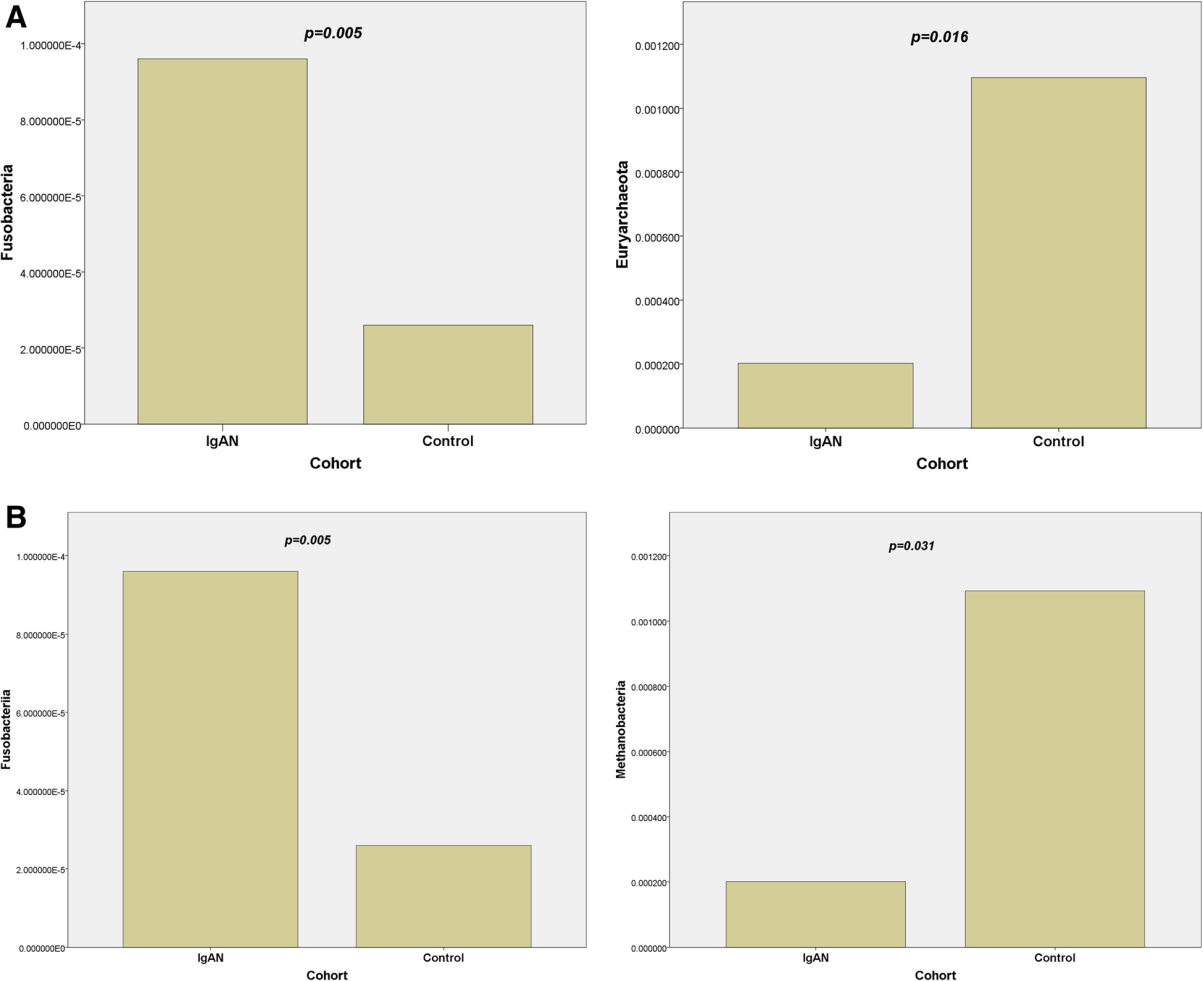
Differences in gut microbiota between IgAN and control cohort. **a** At phylum level (**b**) At class level

At the phylum level, the proportion of *Fusobacteria* was higher in the IgAN cohort (*p* = 0.005) whereas *Euryarchaoeota* was lower in the IgAN cohort (*p* = 0.016) and this is shown in Fig. 3a.

At the class level, *Methanobacteria* was significantly reduced in the IgAN cohort (*p* = 0.031) whereas *Fusobacteriia* (p = 0.005) and *Epsilonproteobacteria* (*p* = 0.018) were increased in the IgAN cohort and shown in Fig. 3b.

Analysing at the genus level, although *Streptococcus* was increased in IgAN group compared to the controls, it was not significant (*p* = 0.432). *Clostridium, Bacillus* and *Lactobacillus*, the mainstay microorganisms in the gut, were reduced in the IgAN group but these too did not reach statistical significance.

**Fig. 4:**
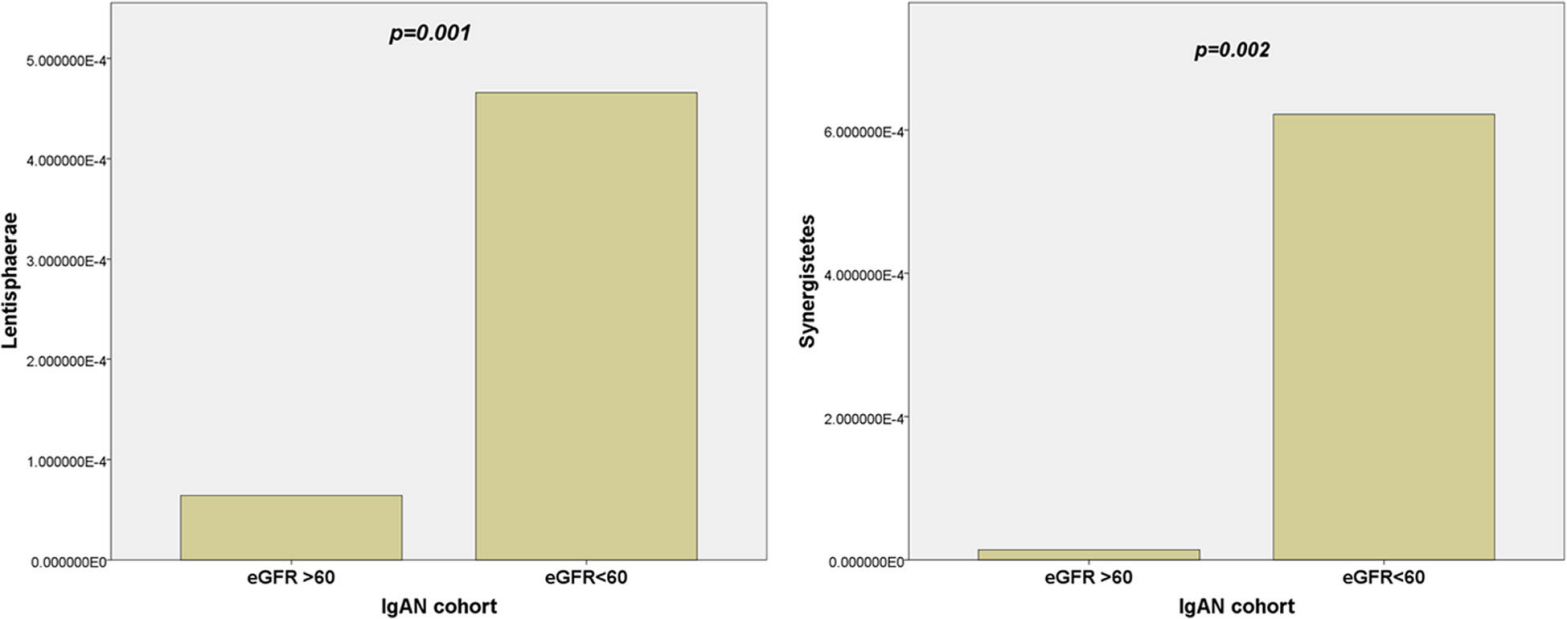
eGFR influencing abundance at the phylum level

On subanalysis of the IgAN cohort, we found that patients with eFGR less than 60 mls/min/1.73m^2^ had an increase in the phyla *Lentisphaerae* (*p* = 0.001) and *Synergistetes* (*p* = 0.002) and displayed in Fig. 4.

## Discussion

Malaysia has a multiracial population and the prevalence of IgAN is 23% with a slight preponderance towards female (1.44:1) [2]. This is lower compared to our neighbouring country, Singapore, where IgAN was reported as high as 43% [37]. However, Malaysian data maybe under-representative of the true incidence due to different screening methods and renal biopsy policies practised.

IgAN poses a substantial risk of progression to ESRD, up to 40% in two decades especially in Asians [31, 38]. Blood pressure reduction and treatment with renin-angiotensin-aldosterone system blockers or immunosuppressant [39] have been advocated to slow the CKD progression although the use of the latter remains controversial with conflicting evidence [40]. In our centre, immunosuppressive therapies are used for patients with heavy proteinuria as Asian studies have shown promising results with steroids [41]. All of our IgAN patients were on either ACE-I or ARB while 64% were on fish oil. 7 out of 12 from the control cohort were on ACE-i/ARB for their hypertension, which was well controlled. The actions of common medications on the gut microbiota cannot be discounted. ACE-i has shown in animal models to improve gut dysbiosis by reducing intestinal permeability, decreasing fibrosis and improving villi length [42]. However, at present, the available data records no obvious changes at phyla level.

Fish oil, rich in polyunsaturated fatty acids, is still widely used as it poses low risk and has a potentially beneficial cardiovascular effects [43]. Fish oil has been studied elaborately to determine its effect on gut microbiota. Some studies have shown no differences in the phyla level [44, 45] while others have shown an increase in Firmicutes and decrease in Bacteroidetes [46–48]. However, none have shown any changes in the Fusobacteria phylum. Both our cohorts did not show significant changes in the Firmicutes and Bacteroidetes phyla.

We decided not to include patients with newly diagnosed who are in active disease or relapsed disease due to several factors. Firstly, our aim is to prove that patients with the disease and in remission are distinguishable from the control group. This theory is needed to confirm that there is fundamental alteration in gut microbiota that plays a role in the pathogenesis of the disease. Secondly, patients with active disease have more variables that results in dysbiosis, − some are clinically ill with ongoing acute kidney injury, while others have hypoalbuminemia which may cause oedema including in the gut. On top of that, in our local practice, patients with active disease are considered for immunosuppressants. All these factors are proven to alter gut microbiota.

Our IgAN cohort was noted to have significantly higher UPCI compared to controls. This is largely due to the fact that these patients have progressed to CKD and have persistent stable proteinuria due to glomerulosclerosis. The eGFR in control group were slightly low as there were patients with CKD stage 3 due to corrected obstructive uropathy. These patients were including to offset the differences of eGFR in between both groups, as CKD severity has already been reported to influence gut microbiota [18].

We discovered no significant difference in OTU richness, gut microbial diversity in IgAN compared to controls. We perceive this could be because all our IgAN patients were in disease remission and not on any immunosuppressive drugs, thus resembling the healthy population. In addition, the assessment of alpha diversity using the Shannon index may have some limitation as shown in other studies [25, 49]. *Bacteroidetes, Firmicutes, Proteobacteria, Verrucomicrobia, Actinobacteria* and *Fusobacteria* constituted > 98% of total abundance and it is similar to other studies worldwide [9, 29, 50, 51]. *Bacteroidetes* and *Firmicutes* monopolise the overall microbiota. *Bacteroidetes *is represented mainly by *Bacteroides* and *Prevotella* whereas the *Firmicutes* consists of mainly the genus *Clostridium*.

The calculated F/B ratio was low in general and reduced further in IgAN cohort. This was due to a decrease in abundance of *Clostridia*. Studies in the European continent have reported a high F/B ratio > 1 [29, 50, 51] whereas the Asian and African continents reported otherwise, < 1 [52, 53]. These differences in gut microbiota profile are likely due to the variation in the diet in which more animal-based protein and fat observed in the Europe diet whereas Asian and African diet is traditionally more plant-based [54]. Significantly greater alpha diversity, increase in OTU and specifically, *Bacteroidetes* phylum were seen with vegans compared to omnivores [55]. These changes in the composition of the microbiome are often due to differences in the amount of directly consumed bacteria, variation in transit time through the intestinal tract, changes in gut pH and regulation of host immunity [56].

The crucial outcome of our study was the significant increase of *Fusobacteria* in the IgAN cohort. *Fusobacteria* has the ability to invade colonic epithelial cells [57]. As such, studies have been conducted to prove its association with inflammatory bowel disease and colon cancer [58]. *Fusobacteria* touted to play a role as an “alpha-bug” which has a virulent capacity to spur remodelling of the entire community, cultivating a pro-inflammatory and pro-carcinogenic response [59]. Could this be the intermediary for abnormal mucosal immunity and gut inflammation that initiates the multi-hit pathogenesis of IgAN?

The *Euryarchaoeota* was significantly lower in the IgAN cohort. This microorganism falls under the domain of Archaea, an obligate anaerobe which is separate from bacteria. They are known to produce methane as a metabolic by-product in hypoxic conditions [60]. There are links between this organism and obesity, constipation and oral cavity infections [61] but we have yet to put this incidental finding in its place.

A unique finding to our study was the increase in alpha diversity with the worsening of CKD. This finding is in contradiction to the general understanding that gut microbial diversity reduces with increasing severity of CKD. Our understanding of this phenomenon is that gut microbiota is predominantly affected by serum urea and toxins such as p-cresol rather than creatinine level [62]. We did not encounter this as there were no significant differences in the urea level between the two groups and the urea level was relatively low in our IgAN cohort. We also report an increase in *Lentisphaerae* (*p* = 0.001) and *Synergistetes* (*p* = 0.002) in patients with eGFR less than 60 mls/min/1.73m^2^. As the relative abundance of these phyla is minute, very few studies have shown their role in our gut ecology.

As IgAN patients often worsen after suffering an upper respiratory tract infection, *Streptococcal* antigens have been implicated with the deposition of IgA in renal tissue [63]. Our findings with regards to *Streptococcus* compared between the two cohorts showed no difference. This may echo with the consensus that the role of tonsillectomy for the purpose of reduction in *Streptococcus* exposure may be limited [36]. Having said that, the role of *Streptococcus* as a gut coloniser may differ in tonsil infection. Moreover, our *Streptococcus* could have been indifferent in both cohorts as our patients were mostly in remission.

Notable discrepancies in our study as compared to that of De Angelis’ et al. [29] would be that our F/B ratio was reduced in the IgAN group whereas the Italians reported an increase. Our *Firmicutes* abundance was reduced in IgAN cohort while *Fusobacteria* and *Actinobacteria* increased in IgAN cohort contrasting the results of the Italian study. Previous studies have also documented a reduction in *Firmicutes* in disease cohorts [62, 64, 65]. Our reduction in Firmicutes is mainly due to reduction in the genus *Clostridium*. *Clostridium* is responsible for the metabolism of carbohydrate to various short-chain fatty acids (SCFA). SCFAs are needed to maintain the intestinal barrier and its reduction will trigger inflammation [66]. We believe that as our IgAN cohort was in prolonged remission, we were unable to achieve statistical significance in the reduction of phyla *Firmicutes* and genus *Clostridium*.

Recently, a new study out of Hunan, China – looking into faecal microbiota characteristics of active IgAN and healthy control, has demonstrated similar findings to ours [67]. They have recorded a reduction in all alpha diversity indexes except Shannon index. Using the Shannon index as the only measurement for alpha diversity has its limitations [68]. In fact, there are still ongoing arguments to justify the best method to evaluate richness diversity, evenness, differentiation and abundance-weighted diversity [69]. Hu et al. also noted a significant increase in the phyla *Fusobacteri*a in their active disease cohort [67]. However, their F/B ratio was higher in the diseased group, concurring with De Angelis et al.

Some notable weaknesses from our study would be our design – cross-sectional study and its sample size. Although the sample size was calculated with type 1 error of 0.05 and power of 80%, a larger-scale study will be needed to confirm these results as gut microbiome has high dimensionality and variability. Another major limitation was the lack of other indexes to evaluate Alpha diversity and Beta diversity. This is mainly due to technical issues we were facing during the time of COVID-19 pandemic. Even then we believe this study is still relevant as it is the only study on IgAN patients who were in remission. Remission state reduces many other confounding variables that may alter the gut microbiome such as patients being unwell during acute illness, hypoalbuminemia, acute kidney injury, as well as a sudden change in medication and diet. Moreover, we contribute to the database of gut microbiota profile for the ASEAN region.

## Conclusion

The richness and abundance of OTU and alpha diversity are unaltered between IgAN patients in remission and controls. Nonetheless, there were significant differences in taxonomic profiling even at the phylum level. Thus, these results might be relevant to understand the basis of microbiota perturbation in the development of IgAN in the Malaysian population. The phyla *Fusobacteria* may be a precursor to intestinal inflammation and autoimmunity in our population. Proving the role of gut dysbiosis as one of the pathogenesis to the development of this disease can facilitate a new population targeted screening, stratification and treatment approach.

## Supplementary Information

**Additional file 1.**

**Additional file 2.**

## Data Availability

The datasets generated during and analysed during the current study is available in the Zenodo repository. DOI: 10.5281/zenodo.4575681
